# LSINCT5 predicts unfavorable prognosis and exerts oncogenic function in osteosarcoma

**DOI:** 10.1042/BSR20190612

**Published:** 2019-05-03

**Authors:** Weidong He, Ming Lu, Dongbo Xiao

**Affiliations:** 1 Department of Joint Surgery, The First People’s Hospital of Lianyungang, Lianyungang 222002, Jiangsu, China; 2 Department of Orthopedics, Medical School of Chinese PLA, Beijing 100853, China; 3 Department of Orthopedics, Ankang Central Hospital, Ankang 725000, Shaanxi, China

**Keywords:** biomarker, large intervening non-coding RNA, LSINCT5, osteosarcoma

## Abstract

The dysregulated expression of LSINCT5 (long stress-induced non-coding transcript 5) has been found in various human tumors, and was generally related to cancer progression and unfavorable prognosis. Although the role of LSINCT5 in osteosarcoma was reported not long ago, the sample size of that study was limited. Our study presented more evidence about the clinical significance and biological function of LSINCT5 in osteosarcoma. In our results, we found LSINCT5 expression was increased in osteosarcoma tissue samples and cell lines, and high LSINCT5 expression was associated with advanced Enneking stage, large tumor size, high histological grade and present distant metastasis. Meanwhile, we observed high LSINCT5 expression was correlated with worse overall survival, and high LSINCT5 expression could be an independent poor predictor for overall survival in osteosarcoma cases. Moreover, we found inhibition of LSINCT5 expression suppressed cell proliferation, migration and invasion *in vitro*, and LSINCT5 overexpression dramatically facilitated cell proliferation, migration and invasion *in vitro*. In conclusion, our study suggests that LSINCT5 exerts oncogenic function in osteosarcoma cells, and may be a potential predictor for clinical outcome in osteosarcoma patients.

## Introduction

Osteosarcoma is the most frequent primary malignant bone tumor, and ranked as the second leading cause of cancer-related deaths among children and adolescents [1]. The character of rapid growth and strong invasiveness are responsible for the poor clinical outcome of patients with osteosarcoma [2,3]. Although the 5-year survival rate of osteosarcoma patients without distant metastasis has risen to over 65% owing to multidisciplinary therapy including surgery, chemotherapy and radiotherapy, the clinical outcome is less than 30% in osteosarcoma patients with distant metastasis, which is obviously poorer than osteosarcoma patients without distant metastasis [4,5]. Therefore, it is essential to find valuable diagnostic or prognostic biomarkers for identifying high risk patients and guiding clinical treatment.

Long non-coding RNAs (lncRNAs) are a diverse class of regulatory RNAs with more than 200 nucleotides in length and limited protein coding capacity [6,7]. LncRNA LSINCT5 (long stress-induced non-coding transcript 5) is a 2.6-kb transcript which is polyadenylated and transcribed from a negative strand between iroquois homeobox (IRX) 4 (IRX4) and IRX2 sites [8]. In recent years, dysregulated expression of LSINCT5 has been found in various human tumors, and was generally related to cancer progression and unfavorable prognosis [9]. Interestingly, LSINCT5 was reported to be overexpressed in osteosarcoma tissues and cells, and inhibition of LSINCT5 significantly depressed osteosarcoma cell proliferation, migration and invasion not long ago [10]. Therefore, our study provided more evidence about the clinical significance and biological function in osteosarcoma. In our study, we also found LSINCT5 expression was increased in osteosarcoma tissues and cells, and obviously correlated with large tumor size, advanced clinical stage and poor prognosis. Moreover, gain-of-function and loss-of-function studies suggested LSINCT5 functioned as oncogenic lncRNA to regulate osteosarcoma cell proliferation, migration and invasion.

## Materials and methods

### Clinical samples

The program of the present study was approved by the Ethics Review Board of The First People’s Hospital of Lianyungang, Chinese PLA General Hospital and Ankang Central Hospital. All patients reviewed and signed informed consent. The 124 osteosarcoma tissues and 35 adjacent normal tissues were collected from 124 patients who received treatment in The First People’s Hospital of Lianyungang, Chinese PLA General Hospital or Ankang Central Hospital. Among 124 osteosarcoma patients, 79 were males and 45 were females, with a median age of 25.4 years (range 10–53 years). All cases had not received any anti-tumor therapy before pathologic diagnosis. Each clinical sample was confirmed by pathologists and then immediately frozen until use.

### RNA isolation and RT-PCR

Total RNAs were extracted from osteosarcoma tissues or cells using TRIzol reagent (Invitrogen, Carlsbad, CA, U.S.A.). Subsequently, cDNA was synthesized by PrimeScript RT Reagent Kit (Takara Biomedical Technology, Beijing, China), and RT-PCR was completed by TB Green Premix ExTaq II (Takara Biomedical Technology, Beijing, China) at Applied Biosystems 7500. The gene-specific primers were as follows: LSINCT5 forward, 5′-TTCGGCAAGCTCCTTTTCTA-3′, and reverse, 5′-GCCCAAGTCCCAAAAAGTTCT-3′; GAPDH forward, 5′-AGCCACATCGCTCAGACAC-3′, and reverse, 5′-GCCCAATACGACCAAATCC-3′. GAPDH acted as the endogenous control for LSINCT5 expression.

### Cell lines and cell transfection

Three osteosarcoma cell lines (HOS, G-292 and Saos-2) and normal osteoblast cell line (hFOB1.19) were cultured in Roswell Park Memorial Institute (RPMI)-1640 medium mixed with the fetal bovine serum (FBS, Gibco, Grand Island, NY, U.S.A.) under humidified atmosphere with 5% CO_2_ at 37°C.

For the knockdown of LSINCT5, the specific siRNA targeting LSINCT5 (siRNA-LSINCT5) and corresponding control siRNA (siRNA-NC) were synthesized by RiboBio Co., Ltd (Guangzhou, China). For up-regulation of LSINCT5, the whole length of LSINCT5 was cloned into pcDNA 3.1 vector (pcDNA-LSINCT5), and the empty vector was as negative control (pcDNA-NC). Cell transfection was performed by Lipofectamine 3000 reagent (Invitrogen, Carlsbad, CA, U.S.A.) based on the manufacturer’s instructions.

### Proliferation assay

The CCK-8 assay (Dojindo Molecular Technologies, Kumamoto, Japan) was used for detecting osteosarcoma cell proliferation ability. Briefly, 1000 cells/well from each cell line were seeded in a 96-well plate. After 24, 48, 72, and 96 h incubation, each well was added with 10 μl of CCK-8 reagent, and cultured for 1 h at 37°C. The optical density (OD) at 450 nm was measured using a microplate monitor.

### Migration and invasion assays

For migration assay, 5 × 10^4^ osteosarcoma cells were suspended in 200-μl serum-free RPMI-1640 medium, and added into the upper chamber. Then, 600 μl RPMI-1640 medium containing 20% FBS was added into the lower chamber. After 24-h incubation, the osteosarcoma cells on the upper membrane surface were removed with cotton, and then the filter membrane was fixed in 4% paraformaldehyde at 4°C for 1 h and stained with 1% Crystal Violet at room temperature for 12 min. The number of migration cells was counted randomly from five visual fields per well by a light microscope. For invasion assay, the upper chamber was pre-coated with 50 μl Matrigel (BD Bioscience, Franklin Lakes, NJ, U.S.A.), and the procedure was analogous to migration assay.

### Statistical analysis

All statistical analyses were conducted through SPSS 22.0 version (Chicago, IL, U.S.A.), and graphed using GraphPad Prism 5.0 version (La Jolla, CA, U.S.A.). All assays were repeated at least in triplicate. Differences between two groups were analyzed using Student’s *t*test. The relationships between LSINCT5 expression and clinicopathological parameters were assessed with Chi-square test. Survival curves were estimated by the Kaplan–Meier method and log-rank test. The prognostic factors were identified by univariate and multivariate Cox regression analyses. A *P*-value less than 0.05 was considered statistically significant.

## Results

### LSINCT5 is identified as an up-regulated lncRNA in osteosarcoma

**Figure 1:**
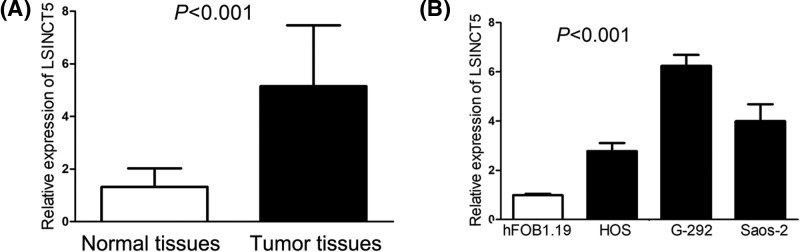
LSINCT5 is identified as an up-regulated lncRNA in osteosarcoma (**A**) LSINCT5 expression was markedly increased in osteosarcoma tissues compared with adjacent normal tissues. (**B**) LSINCT5 expression in osteosarcoma cell lines was higher than normal osteoblast cell line.

To identify LSINCT5 expression in osteosarcoma, we performed RT-PCR to detect LSINCT5 expression levels in osteosarcoma tissues and cell lines (HOS, G-292 and Saos-2), adjacent normal tissues and normal osteoblast cell line (hFOB1.19). We found LSINCT5 expression was markedly increased in osteosarcoma tissues compared with adjacent normal tissues (Figure 1A). Meanwhile, osteosarcoma cell lines (HOS, G-292 and Saos-2) also showed higher levels of LSINCT5 expression than normal osteoblast cell line (hFOB1.19) (Figure 1B).

### Correlation between LSINCT5 expression and clinical features in osteosarcoma

In order to know the clinical significance of LSINCT5 in osteosarcoma cases, all osteosarcoma cases in our study were divided into two groups (high LSINCT5 expression group and low LSINCT5 expression group) by using the median value of LSINCT5 expression level as the cut-off value. In correlation analysis (Table 1), the high LSINCT5 expression was proved to be correlated with advanced Enneking stage, large tumor size, high histological grade and present distant metastasis. However, LSINCT5 expression had no significant correlation with gender, age and tumor site in osteosarcoma cases.

**Table 1:** Associtations between LSINCT5 expression and clinicopathological characteristics in osteosarcoma patients

Characteristics	*n*	High expression (%)	Low expression (%)	*P*
Age (years)				
≤18	49	27 (55.1)	22 (44.9)	0.358
>18	75	35 (46.7)	40 (53.3)	
Gender				
Female	45	22 (44.9)	27 (55.1)	0.358
Male	79	40 (53.3)	35 (46.7)	
Enneking stage				
I-II A	44	15 (34.1)	29 (65.9)	0.009
II B-III	80	47 (58.8)	33 (41.3)	
Tumor size				
≤8 cm	71	29 (40.8)	42 (59.2)	0.018
>8 cm	53	33 (62.3)	20 (37.7)	
Distant metastasis				
Absence	102	44 (43.1)	58 (56.9)	0.001
Presence	22	18 (81.8)	4 (18.2)	
Histological grade				
G1-G2	55	21 (38.2)	34 (61.8)	0.019
G3-G4	69	41 (59.4)	28 (40.6)	
Tumor site				
Femur/Tibia	99	47 (47.5)	52 (52.5)	0.263
Other	25	15 (60.0)	10 (40.0)	

### Correlation between LSINCT5 expression and clinical outcome in osteosarcoma

**Figure 2:**
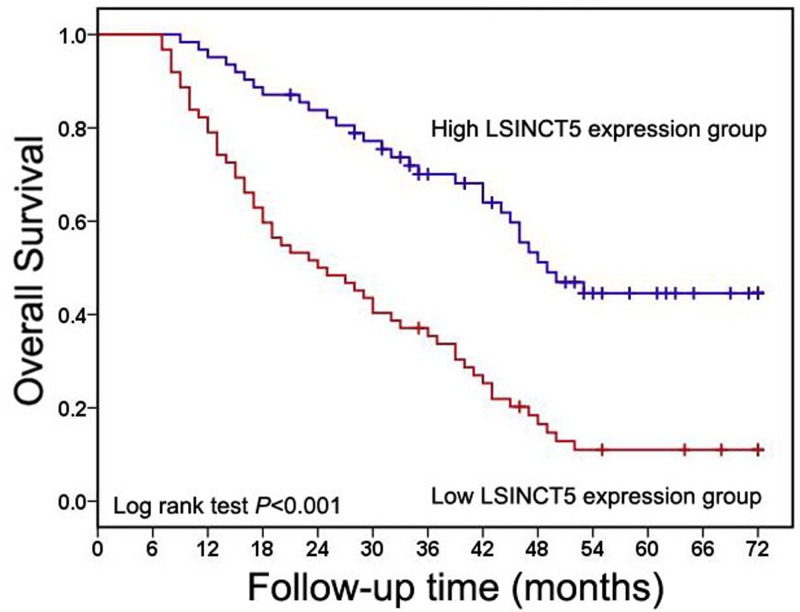
High LSINCT5 expression predicts poor overall survival in osteosarcoma patients Kaplan–Meier method and log-rank tests was used to estimate the effect of LSINCT5 expression level on the overall survival time in osteosarcoma patients.

To further explore the prognostic significance of LSINCT5 expression in osteosarcoma cases, we used Kaplan–Meier method and log-rank tests to estimate the effect of LSINCT5 expression level on the overall survival time. As shown in Figure 2, osteosarcoma patients with high levels of LSINCT5 expression showed worse overall survival time than those with low levels of LSINCT5 expression. Moreover, results of univariate and multivariate Cox regression analyses further suggested that high LSINCT5 expression could be an independent poor predictor for overall survival in osteosarcoma cases (Table 2).

**Table 2:** Univariate and multivariate Cox regression of prognostic factors for overall survival in osteosarcoma patients

Parameter	Univariate analysis			Multivariate analysis		
	HR	95% CI	*P*	HR	95% CI	*P*
Age (years)						
(≤18 vs. >18)	0.842	0.547–1.296	0.435			
Gender						
(Female vs. Male)	1.145	0.732–1.791	0.553			
Enneking stage						
(I-III A vs. II B-III)	3.723	2.176–6.370	<0.001	1.898	0.874–4.124	0.047
Tumor size						
(≤8 vs. >>8 cm)	1.880	1.223–2.892	0.004	1.358	0.868–2.124	0.180
Distant metastasis						
(Absence vs. Presence)	5.611	3.230–9.748	<0.001	3.152	1.748–5.684	<0.001
Histological grade						
(G1-G2 vs.G3-G4)	2.754	1.722–4.405	<0.001	1.388	0.729–2.644	0.319
Tumor site						
(Femur/Tibia vs. Other)	1.612	0.980–2.652	0.060			
LSINCT5 expression						
(Low vs. High)	2.925	1.864–4.591	<0.001	1.675	1.017–2.759	0.043

Abbreviations: HR, hazard ratio; 95% CI, 95% confidence interval.

### The effect of LSINCT5 on osteosarcoma cell proliferation, migration and invasion

**Figure 3:**
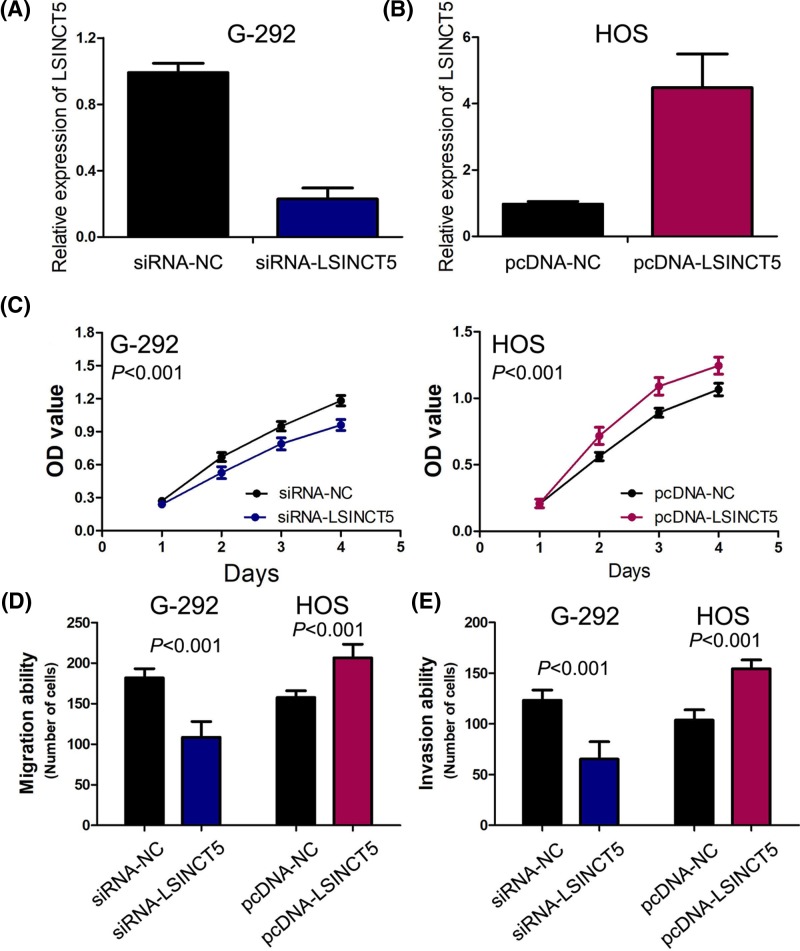
LSINCT5 functions as oncogenic lncRNA to modulate osteosarcoma cell proliferation, migration and invasion (**A**) siRNA-LSINCT5 decreased LSINCT5 expression in G-292 cells. (**B**) pcDNA-LSINCT5 increased LSINCT5 expression in HOS cells. (**C**) Inhibition of LSINCT5 expression attenuated cell viability in G-292cells, and LSINCT5 overexpression remarkably enhanced cell viability in HOS cells. (**D**) Inhibition of LSINCT5 expression suppressed cell migration in G-292 cells, and LSINCT5 overexpression facilitated cell migration in HOS cells. (**E**) Inhibition of LSINCT5 expression depressed cell invasion in G-292cells, and LSINCT5 overexpression enhanced cell invasion in HOS cells.

To gain insight into the biological functional of LSINCT5 on osteosarcoma cells, we observed the LSINCT5 expression levels in osteosarcoma tissues and cell lines (HOS, G-292 and Saos-2), and chose G-292 cells for loss-of-function study and HOS cells for gain-of-function study. The transduction efficiencies were detected by RT-PCR, siRNA-LSINCT5 decreased LSINCT5 expression by as much as 20% in G-292 cells (Figure 3A), and pcDNA-LSINCT5 increased LSINCT5 expression approximately six times in HOS cells (Figure 3B). Subsequent CCK-8 assays showed that inhibition of LSINCT5 expression obviously attenuated cell viability in G-292cells, and LSINCT5 overexpression remarkably enhanced cell viability in HOS cells (Figure 3C). Moreover, migration and invasion assays demonstrated that inhibition of LSINCT5 expression definitely suppressed cell migration and invasion in G-292cells, and LSINCT5 overexpression dramatically facilitated cell migration and invasion in HOS cells (Figure 3D,E).

## Discussion

LSINCT5 is a stress-induced long noncoding transcript with a length of 2.6 kb. Originally, Silva et al. [11] performed whole-genome tiling arrays to analyze the transcription across the entire genome in both normal human bronchial epithelial cells and the cells exposed to the tobacco carcinogen, and identified 12 long non-coding transcripts which were named LSINCTs. Subsequently, Silva et al. [12] further found LSINCT5 expression levels were obviously increased in breast and ovarian cancer tissues and cell lines compared with their corresponding normal tissues and cell lines, respectively. Recently, LSINCT5 overexpression has been identified in lung cancer [13], hepatocellular carcinoma [14], bladder cancer [15], gastric cancer [16,17] and colorectal cancer [16]. In our study, we also found LSINCT5 expression was increased in osteosarcoma tissue samples and cell lines, which was similar to recent study reported by Kong et al. [10]. In addition, Kong et al. [10] further analyzed the relationship between LSINCT5 expression and clinicopathological characteristics in 42 osteosarcoma cases, and found high LSINCT5 expression was associated with advanced TNM stage, large tumor size and present metastasis. In our study, we further explored the clinical value of LSINCT5 expression in 124 osteosarcoma cases, and also observed that high LSINCT5 expression had significant correlations with advanced Enneking stage, large tumor size, high histological grade and present distant metastasis. Generally, more studies were still need to confirmed the clinical significance of LSINCT5 in osteosarcoma patients. Besides, Tian et al. [13] showed levels of LSINCT5 expression were related to advanced TNM stages, tumor size and positive metastasis. In hepatocellular carcinoma, Li et al. [14] reported patients with advanced clinical stage or positive metastasis had significantly increasing LSINCT5 expression compared with patients with early clinical stage or negative metastasis. Moreover, the positive correlation between LSINCT5 expression and clinical stage was also observed in bladder cancer patients by Zhu et al. [15]. In gastric cancer patients, Xu et al. [16] suggested LSINCT5 overexpression was correlated with the presence of large tumor size, deep tumor invasion depth, lymphatic metastasis and advanced TNM stage. Meanwhile, Xu et al. [16] also showed high LSINCT5 expression was associated with large tumor size, deep tumor invasion depth and advanced TNM stage in colorectal cancer patients. Long et al. [18] demonstrated that ovarian cancer patients with high LSINCT5 expression tended to have advanced FIGO stage and lymph node metastasis. However, Mansoori et al. [19] indicated there was no significant correlation between LSINCT5 expression and clinicopathological characteristics in breast cancer patients.

The prognostic value of LSINCT5 has been reported in osteosarcoma [10], hepatocellular carcinoma [14], bladder cancer [15], gastric cancer [16] and colorectal cancer [16]. In osteosarcoma patients, Kong et al. [10] conducted Kaplan–Meier method and log-rank test to explore the correlation between LSINCT5 expression and clinical outcome, and found high LSINCT5 expression was correlated with worse clinical outcome. Similarly, the results of survival analysis also showed high LSINCT5 expression was associated with short overall survival in osteosarcoma patients. In addition, we further conducted univariate and multivariate Cox regression analyses to identify independent prognostic factors, and found high LSINCT5 expression could be an independent poor predictor for overall survival in osteosarcoma cases. Besides, Li et al. [14] revealed that high levels of LSINCT5 expression predicted unfavorable prognosis in hepatocellular carcinoma patients. In bladder cancer cases, Zhu et al. [15] suggested patients with high LSINCT5 expression had shorter overall survival than those with low LSINCT5 expression. Moreover, Xu et al. showed LSINCT5 overexpression was associated with short disease-free survival [16] and disease-specific survival in gastric cancer and colorectal cancer patients.

LSINCT5 has been suggested to function as oncogenic lncRNA to affect tumor behavior in several human cancers including osteosarcoma. Recently, Kong et al. [10] performed loss-of-function study, and found down-regulation of LSINCT5 depressed osteosarcoma cell proliferation *in vitro* and growth *in vivo*. In our study, we conducted loss-of-function study and gain-of-function study, and also found inhibition of LSINCT5 expression suppressed cell proliferation, migration and invasion *in vitro*, and LSINCT5 overexpression dramatically facilitated cell proliferation, migration and invasion *in vitro*. Unfortunately, the limit of our study lacked of research on molecular mechanisms of LSINCT5 in osteosarcoma cells due to inadequate research funds. However, Kong et al. [10] found LSINCT5 modulated EZH2 to suppress APC expression and activate the Wnt/β-catenin pathway in in osteosarcoma cells.

In conclusion, high LSINCT5 expression is correlated with clinical progression and unfavorable prognosis. LSINCT5 functions as oncogenic lncRNA to modulate osteosarcoma cell proliferation, migration and invasion.

## Abbreviations

CCK-8: cell counting kit-8
EZH2: enhancer of zeste 2 polycomb repressive complex 2 subunit
FBS: fetal bovine serum
FIGO: international federation of gynecology and obstetrics
GADPH: glyceraldehyde-3-phosphate dehydrogenase
IRX: iroquois homeobox
lncRNA: long non-coding RNA
LSINCT5: long stress-induced non-coding transcript 5
RPMI: Roswell Park Memorial Institute
TNM: tumor node metastasis
